# Complementary and alternative medicine use by visitors to rural Japanese family medicine clinics: results from the international complementary and alternative medicine survey

**DOI:** 10.1186/1472-6882-14-360

**Published:** 2014-09-25

**Authors:** Gregory Shumer, Sara Warber, Satoko Motohara, Ayaka Yajima, Melissa Plegue, Matthew Bialko, Tomoko Iida, Kiyoshi Sano, Masaki Amenomori, Tsukasa Tsuda, Michael D Fetters

**Affiliations:** Family Medicine Department, Chelsea Family Medicine Center, University of Michigan, 14700 E. Old U.S. Hwy. 12, Chelsea, MI 48118 UK; Family Medicine Department, University of Michigan, 1018 Fuller Street, Ann Arbor, MI 48104-1213 USA; Kikugawa Home Medical Center, 1055-1 Kikugawa, Kikugawa City, Shizuoka Prefecture, Japan; Haibara General Hospital, 2887-1 Hosoe, Makinohara City, Shizuoka Prefecture, Japan; Yuge Family Medicine Clinic, 1825 Yuge, Ryuo Town, Shiga Prefecture, Japan

**Keywords:** Complementary and Alternative Medicine (CAM), Integrative medicine, Primary care, Family medicine, Japan, International Complementary and Alternative Medicine Questionnaire (I-CAM-Q)

## Abstract

**Background:**

There is growing interest in the use of complementary and alternative medicine (CAM) throughout the world, however previous research done in Japan has focused primarily on CAM use in major cities. The purpose of this study was to develop and distribute a Japanese version of the International Complementary and Alternative Medicine Questionnaire (I-CAM-Q) to assess the use of CAM among people who visit rural Japanese family medicine clinics.

**Methods:**

Using a Japanese version of the International Complementary and Alternative Medicine Questionnaire (I-CAM-Q), a cross-sectional survey was conducted in three rural family medicine clinics. All patients and those accompanying patients who met inclusion criteria were eligible to participate. Data were entered into SPSS Statistics and analyzed for use by age, gender, and location.

**Results:**

Of the 519 respondents who participated in the project, 415 participants reported CAM use in the past 12 months (80.0%). When prayer is excluded, the prevalence of CAM use drops to 77.3% in the past year, or 403 respondents. The most common forms of CAM used by respondents were pain relief pads (n = 170, 32.8%), herbal medicines/supplements (n = 167, 32.2%), and massage by self or family (n = 166, 32.0%). Female respondents, individuals with higher levels of education, and those with poorer overall health status were more likely to use CAM than respondents without these characteristics. Only 22.8% of CAM therapies used were reported to physicians by survey participants.

**Conclusions:**

These data indicate that CAM use in rural Japan is common. The results are consistent with previous studies that show that Japanese individuals are more interested in forms of CAM such as pain relief pads and massage, than in mind-body forms of CAM like relaxation and meditation. Due to the high utilization of certain CAM practices, and given that most CAM users do not disclose their CAM use to their doctors, we conclude that physicians in rural Japan would benefit by asking about CAM use during patient interviews, and by familiarizing themselves with the potential benefits and risks of commonly used CAM modalities.

**Electronic supplementary material:**

The online version of this article (doi:10.1186/1472-6882-14-360) contains supplementary material, which is available to authorized users.

## Background

Complementary and alternative medicine (CAM) is defined by the National Center for Complementary and Alternative Medicine (NCCAM) as “a group of diverse medical and health care systems, practices, and products that are not considered part of conventional medicine” [1]. Examples of commonly used types of CAM include manual medicine such as massage therapy and spinal manipulation, traditional Chinese medicine such as tai chi, qi gong, and Chinese herbal medicine, and mind-body medicine such as mindfulness meditation, guided imagery, and relaxation techniques [1].

The use of CAM modalities is common in certain populations, and gaining popularity in others [2]. While CAM use is common, it has proven to be difficult to compare across populations due to varying study designs and different definitions of CAM [3, 4]. For example, when exercise and prayer are included in the definition of CAM, the prevalence of CAM use not surprisingly increases dramatically [4]. However, most research studies do not include prayer and exercise in the definition of CAM. The percentage of people using at least one form of CAM in cross-sectional nation-wide studies that did not include prayer or exercise in their definitions of CAM was found to be 26.3% in the UK in 2005, 38.3% in the USA in 2007, 42.3% in Germany in 2008, and 68.9% in Australia in 2005 [5–8]. Available data also suggests that CAM use is more prevalent in rural areas than in urban areas [3].

While the use of CAM is common in multiple populations, previous research also shows that patients who use CAM commonly do not communicate this use with their primary care providers [2, 9–11]. In order to understand the scope of this problem, it is necessary to quantify the use of CAM in various populations. In 2006, an international workshop was held in Norway to create a standardized survey quantifying the use of CAM, and the resulting instrument is the International Complementary and Alternative Medicine Questionnaire (I-CAM-Q) [12]. A key rationale for the development of the survey is to have a standardized approach to measuring CAM use that will allow comparisons among various populations and countries.

Use of CAM in Japan is of particular interest because several well-known types of CAM, such as herbal medicine – known in Japan as kampo – and acupuncture, have existed there for hundreds of years, and have experienced a recent revival in popularity and use [13–15]. Moreover, there is a higher level of integration between CAM and conventional medicine in Japan than in most developed countries, with modern Western medicine often coexisting with certain types of traditional Asian medicine, such as kampo and acupuncture, that are commonly classified as CAM [14]. Specifically, kampo can be prescribed by physicians, and has been covered by the public health insurance system in Japan since 1976 [15]. Kampo use is common – a study in 1999 conducted by interviews with Japanese physicians found that 70% integrated the use of kampo into their patient care [16].

Given the apparent high frequency of kampo prescribing by Japanese physicians, previous researchers have explored the prevalence of CAM education – with a specific focus on kampo education - in Japanese medical schools. One study found that 86% of Japanese medical schools include kampo in their pharmacology curriculum, and 17% include broader CAM education beyond kampo [17]. A 2012 study surveyed all 80 Japanese medical schools, and found that 98% of schools offered at least one kampo course, 84% offered four or more kampo courses, and 29% employed full-time kampo medicine instructors [18]. A survey of physician graduates from Jichi Medical University supports the notion that kampo is commonly prescribed by Japanese physicians, with 30% of respondents prescribing kampo formulae daily, 45% occasionally, 22% rarely, and only 3% never [19].

Despite the historical origins of certain types of CAM, coverage under the national health insurance system since 1976, and the high frequency of CAM prescribing by physicians, there has been little research done on the prevalence of actual CAM use by patients in Japan. One large, nationwide study examined CAM use in the context of oncology patients, and indicated that 44.6% of cancer patients used CAM, with the majority (96.2%) using products such as mushrooms, herbs, and shark cartilage, and most (60.7%) using CAM without consulting a physician [20].

To date there have been only two studies about CAM use in general populations in Japan, one through a telephone survey of the general population, and the other a survey in urban clinics. The nation-wide telephone survey conducted in 2001 showed that 76.0% of respondents had used at least one type of CAM in the past 12 months, with the most commonly used modalities being nutritional and tonic drinks (43.1%), dietary supplements (43.1%), health-related appliances (21.5%), herbs or over-the-counter kampo (17.2%), and massage or acupressure (14.8%) [13]. In the 2002 study in Tokyo outpatient clinics, 49.6% of participants had used at least one type of CAM in the past 12 months, with the most common types being massage (21.4%), vitamins (17.1%), health foods and dietary supplements (11.3%), acupressure (10.3%), and kampo (9.3%) [21].

While it appears that CAM is commonly utilized in Japan, no study has focused on its use in rural Japanese settings. The purpose of this study was to develop and distribute a Japanese version of the I-CAM-Q to assess the use of CAM among people who visit rural Japanese family medicine clinics.

## Methods

### Design

The study design employed an anonymous, cross-sectional survey distributed to patients and visitors in family medicine clinic waiting areas in three sites over a total period of six weeks. The University of Michigan Institutional Review Board (IRBMED) reviewed the study design, and exempted the study from IRB review based on federal exemption #2 of the 45 CFR 46.101 (b) [Additional file 1]. The study was accepted by local hospital IRB at two of the clinic sites in Kikugawa and Mori-machi, and by the clinic director at the third site in Yuge.

### Setting and population

The research setting was three family medicine clinics in rural Japan located in Shizuoka and Shiga prefectures. The three clinic sites in Kikugawa, Mori-machi, and Yuge were chosen because of their affiliation with the University of Michigan Family Medicine Department, their location in rural Japanese communities, and their willingness to be a part of this research study. Kikugawa is an agricultural city that is known for green tea production, located in the western portion of Shizuoka prefecture. Mori-machi is a small inland town located in Shizuoka prefecture. Yuge is a small village that has merged to become a part of the larger Ryuo town, which is an industrial and agricultural locale near the center of Shiga prefecture. At the time of data collection, there were nine physicians (two attendings and seven residents) working at the Mori-machi clinic, 11 physicians (three attendings, two clinical fellows, and six residents) working at the Kikugawa clinic, and seven physicians (four full-time physicians, three part-time physicians) working at the Yuge clinic.

Data collection covered six weeks from January 14th-February 22nd, 2013, with two weeks of data collection at each site. On average, data was collected on four days during each week. All patients and visitors who met inclusion criteria were invited to participate using a standardized recruitment script in Japanese developed by the research team. Participation was voluntary. Inclusion criteria required that participants be 20 years of age or older, speak and read Japanese, and provide verbal consent to participate in the study. Exclusion criteria included patients and visitors who were under 20 years of age, and those with debilitating diseases that precluded meaningful participation.

### Survey instrument

The I-CAM-Q is meant to be self administered, and adaptable for different target populations. The original I-CAM-Q contains four sections: 1) visits to various healthcare providers, 2) complementary treatments received from physicians, 3) self-help practices, and 4) use of herbal medicine and dietary supplements [12].

The study required sociocultural adaptation of the I-CAM-Q, and translation into Japanese. An initial meeting was held with members of the primary research team to decide the best way to appropriately adapt the original I-CAM-Q for use with a rural Japanese population. The original structure of the I-CAM-Q was maintained, but changes were made to include commonly used CAM modalities in Japan [Additional file 2]. An explanatory face sheet and demographics page were also created and added to the survey [Additional files 3 and 4].

After creating the survey documents in English, two native-speaking Japanese research members (SM and AY) independently translated the adapted instrument into Japanese. After independent translation was complete, they compared the translations item-by-item, and agreed upon the best translation. The Japanese instrument was then cognitively tested on 10 native Japanese speakers to assess comprehension of the content as intended by the investigators, and minor refinements were made based on their comments. As a final preparatory step, the instrument, along with explanatory face sheet and demographics page, were sent to six family medicine physicians at the three target sites in Japan, and minor changes were made based on their expertise and feedback [Additional files 5, 6, and 7].

### Recruitment

During the study period, patients and visitors in the waiting rooms of the selected three clinics who met inclusion criteria were approached by this study’s first author and invited using the standardized recruitment script to complete the survey. Those who chose to participate were instructed to place their surveys in a collection box in the clinic after completion.

### Data entry and analysis

Completed surveys were entered daily into SPSS (Statistical Package for Social Sciences). All free-text responses were transliterated phonetically from Japanese into English and entered into the SPSS. One survey from each day of collection was selected by a random number generator to be saved for validity testing and data entry accuracy. Validity testing was completed after data collection, with over 97% agreement between initial data entry and subsequent data entry of saved surveys by two separate authors (SM and AY).

Three researchers (SW, SM, and AY) individually and independently categorized each of the free-written responses from the herbal medicines and dietary supplements section into one of the following categories: 1) kampo, 2) vitamins, 3) energy and nutritional drinks, 4) other herbs, 5) other supplements, and 6) dropped. Items were dropped if they did not fit into any of the categories. The three study authors then met to review categorization and establish consensus for each entry. Data were analyzed, using chi-squared tests, to look at CAM use by category, age, gender, self-reported health rating, self-reported health problems, education, and clinic site.

## Results

### Demographics

Overall, 649 individuals were approached and 519 agreed to participate in the study (80.0% response rate). Table 1 provides the overall demographics of all respondents. The average age of survey participants was 50 (SD = 16.5), with 65.8% female respondents and 34.2% male respondents among those with valid gender identification. The majority of participants reported having at least one type of health problem in the past year, with the most common problems being musculoskeletal (40.7%), psychological (24.9%), gastrointestinal (25.4%), pulmonary (23.7%), and cardiovascular (18.1%).

**Table 1:** **Demographics**

Variable	Frequency (%)
**Site**	
Kikugawa	181 (34.9)
Mori-machi	173 (33.3)
Yuge	165 (31.8)
**Gender**	
Male	151 (29.1)
Female	290 (55.9)
No answer	78 (15.0)
**Rate health**	
Excellent	19 (3.7)
Good	112 (21.6)
Fair	241 (46.4)
Poor	65 (12.5)
No answer	82 (15.8)
**Health problems**	
Musculoskeletal	211 (40.7)
Gastrointestinal	132 (25.4)
Mental and psychological	129 (24.9)
Pulmonary	123 (23.7)
Cardiovascular	94 (18.1)
Allergies	66 (12.7)
Gynecologic	57 (11.0)
Neurologic	56 (10.8)
Skin	48 (9.2)
Urologic	37 (7.1)
Chronic pain	32 (6.2)
Endocrine	28 (5.4)
Cancer	8 (1.5)
Kidney	6 (1.2)
Other	31 (6.0)
**Number of health problems – Mean (SD)**	2.04 (1.9)
**Age – Mean (SD), n = 440**	49.86 (16.5)

Overall, 80.0% of respondents reported using at least one form of CAM in the past 12 months, and only 22.8% of CAM therapies were self-reported to physicians. The 80.0% prevalence value includes any person who responded that they had used any of the products, providers, or treatments listed in Tables 2, 3, 4, and 5, except for “physician” in Table 4. When prayer is also excluded from our study – by excluding positive answers to “praying for own health” and “attending traditional healing ceremonies (temples, shrines, etc.) for health” in Table 2 – the number of respondents using at least one form of CAM in the past 12 months drops to 403, or 77.6% of respondents.

**Table 2:** **Self-help practices**

Self-help practices used in the past year	Used (n, (%))**	Motivation*				Helpfulness* yes or somewhat (%)	% Who tell doctor*
		Acute illness (%)	Long-term illness (%)	Health maintenance (%)	Other (%)		
Pain relief pads	170 (32.8%)	37.1	33.8	12.6	16.6	86.6	26.7
Massage (by self or family)	166 (32.0%)	21.0	29.4	33.6	16.1	87.4	12.6
Praying for own health	95 (18.3%)	7.7	32.1	46.2	14.1	45.9	1.5
Attending traditional healing ceremony (temples, shrines, etc.) for health	87 (16.8%)	1.2	32.1	50.6	16.0	28.6	1.6
Massage device	85 (16.4%)	11.0	26.0	45.2	17.8	79.7	16.9
Dietary therapy	58 (11.2%)	3.9	39.2	52.9	3.9	84.1	41.0
Wearing talisman for health or recovery	56 (10.8%)	4.5	29.5	47.7	18.2	50.0	5.7
Electrotherapy device (not massage)	48 (9.2%)	19.0	31.0	38.1	11.9	78.0	17.6
Hot-spring therapy	41 (7.9%)	8.1	21.6	62.2	8.1	76.3	9.4
Yoga	28 (5.4%)	0	3.8	84.6	11.5	72.0	9.1
Aromatherapy	25 (4.8%)	0	30.4	34.8	34.8	85.7	5.6
Tai Chi/Qigong	5 (1.0%)	25.0	25.0	25.0	25.0	100	33.3
Moxibustion	4 (0.8%)	25.0	50.0	25.0	0	75.0	0
Zen/meditation	4 (0.8%)	0	50.0	0	50.0	66.7	0
Cupping	3 (0.6%)	0	33.3	0	66.7	100	100
Other	7 (1.3%)	16.7	50.0	16.7	16.7	100	66.7

*Valid percentages are reported; when a participant indicates visitation or use, they are asked but not required, to answer subsequent questions on motivation, helpfulness, and whether or not they told their physician about use (sometimes resulting in different n-value).

**Denominator is total study respondent number of 519.

**Table 3:** **Type of CAM Products used by Individuals**

Type of CAM product	Number (%) of products used by respondents*
Other supplement**	82 (31.2%)
Kampo	77 (29.3%)
Energy or nutritional drink	38 (14.4%)
Vitamin	30 (11.4%)
Other Herb	12 (4.6%)
Dropped***	24 (9.1%)

*Denominator is 263, the total number of products listed by respondents.

**Examples of other supplements include calcium, iron, omega 3, and mixtures.

***Reason for dropping: pharmaceutical (n = 11), topical (n = 7), food (n = 2), inadequate information (n = 2), not applicable (n = 2).

**Table 4:** **Healthcare Providers seen**

Healthcare providers seen in the past year	Visited n, (%)**	Motivation*				Helpfulness* very or somewhat (%)	% Who tell doctor*
		Acute illness (%)	Longterm illness (%)	Health maintenance (%)	Other (%)		
Physician	366 (70.5%)	25.5	40.9	16.2	17.4	95.5	N/A
Massage Therapist	39 (7.5%)	17.1	28.6	45.7	8.6	77.1	20.0
Chiropractor	35 (6.7%)	14.7	32.4	44.1	8.8	81.2	33.3
Acupuncturist	24 (4.6%)	13.6	31.9	36.4	18.9	81.0	33.3
Bonesetter***	21 (4.0%)	25.0	40.0	20.0	15.0	73.7	53.8
Rehabilitation therapist	12 (2.3%)	9.1	54.5	18.2	18.2	100	70.0
Spiritual healer	5 (1.0%)	0	20.0	20.0	60.0	100	40.0
Qigong therapist	5 (1.0%)	0	40.0	20.0	40.0	66.7	0
Kampo practitioner	5 (1.0%)	25.0	25.0	50.0	0	100	40.0
Other	6 (1.2%)	0	50.0	33.3	16.7	100	0

*Valid percentages are reported; when a participant indicates visitation or use, they are asked but not required, to answer subsequent questions on motivation, helpfulness, and whether or not they told their physician about use (sometimes resulting in different n-value).

**Denominator is total study respondent number of 519.

***Bonesetters are practitioners of joint manipulation.

**Table 5:** **Alternative Treatments received from Physicians**

Alternative treatments received in the past year	Yes n, (%)**	Motivation* (%)				Helpfulness* yes or somewhat (%)
		Acute illness	Long-term illness	Health maintenance	Other	
Kampo	47 (9.1%)	27.3	52.3	13.6	6.8	79.5
Supplements	18 (3.5%)	6.7	26.7	26.7	40.0	80.0
Acupuncture	12 (2.3%)	9.1	54.5	18.2	18.2	80.0
Other	9 (1.7%)	11.1	55.6	33.3	0	100

*Valid percentages are reported; when a participant indicates visitation or use, they are asked but not required, to answer subsequent questions on motivation, helpfulness, and whether or not they told their physician about use (sometimes resulting in different n-value).

**Denominator is total study respondent number of 519.

Differences in CAM use amongst subgroups were assessed. There were no significant differences in CAM use across clinic sites. Gender was significant in two categories, with females more likely to use self-help CAM methods (p = 0.002, 76.2% of women vs. 62.2% of men) and CAM products (p < 0.001, 42.1% vs. 24.5%) than males. The use of CAM products also had a significant association with education, with users of CAM products tending to have higher educations – 46.4% of respondents with some college or more used CAM products, while only 28.5% of respondents with high school or less used CAM products (p < 0.001). For health ratings, more individuals who rated themselves as having ‘poor’ health reported seeing CAM providers (p = 0.004, 33.8% vs. 18.3%) and receiving CAM treatments (p < 0.001, 32.3% vs. 11.8%) than all other respondents.

### Self-help CAM and CAM products

Table 2 illustrates the respondents’ use of self-help CAM modalities. Self-help practice was the most commonly used form of CAM, with 367 respondents (70.7%) reporting use of at least one self-help therapy. When prayer and attending temples and shrines are removed from this category, 344 respondents (66.3%) reported use of at least one therapy. Pain relief pads (n = 170, 32.8%) and massage by self or family (n = 166, 32.0%) were the most commonly used modalities. Nine other self-help modalities were used by at least 5% of participants in the past year. For every self-help practice except dietary therapy, less than 30.0% of respondents who reported use of these practices revealed their use to their primary care physicians. The most common primary motivations for using self-help practices were for treatment of long-term illnesses or for health maintenance, and most respondents found these practices very- or somewhat- helpful. Respondents who reported musculoskeletal problems were significantly more likely to use self-help practices than other respondents, as displayed in Table 6.

**Table 6:** **Associations between Health Problems and CAM usage**

Health problems		Total sample, n = 519	Using any form of CAM, n = 415 (80.0%)	Seeing a CAM provider, n = 111 (21.4%)	Receiving CAM treatment, n = 77 (14.8%)	Using CAM self-help, n = 367 (70.7%)	Using CAM product, n = 168 (32.4%)
Musculoskeletal	Present	n = 211	194 (91.9)*	64 (30.3)*	35 (16.6)	179 (84.8)*	93 (44.1)*
	Absent	n = 308	**221 (71.8)**	**47 (15.3)**	42 (13.6)	**188 (61.0)**	**75 (24.4)**
Cardiovascular	Present	n = 94	73 (77.7)	17 (18.1)	18 (19.1)	61 (64.9)	29 (30.9)
	Absent	n = 425	342 (80.5)	94 (22.1)	59 (13.9)	306 (72.0)	139 (32.7)
Pulmonary	Present	n = 123	106 (86.2)	34 (27.6)	28 (22.8)	96 (78.0)	60 (48.8)
	Absent	n = 396	309 (78.0)	77 (19.4)	49 (12.4)	271 (68.4)	108 (27.3)
Neurologic	Present	n = 56	49 (87.5)	23 (41.1)*	16 (28.6)	45 (80.4)	21 (37.5)
	Absent	n = 463	366 (79.0)	**88 (19.0)**	61 (13.2)	322 (69.5)	147 (31.7)
Gastrointestinal	Present	n = 132	110 (83.3)	32 (24.2)	29 (22.0)	100 (75.8)	65 (49.2)*
	Absent	n = 387	305 (78.8)	79 (20.4)	48 (12.4)	267 (69.0)	**103 (26.6)**
Gynecologic**	Present	n = 57	54 (94.7)	18 (31.6)	17 (29.8)	48 (84.2)	32 (56.1)
	Absent	n = 233	187 (80.3)	46 (19.7)	29 (12.4)	173 (74.2)	90 (38.6)
Allergies	Present	n = 66	59 (89.4)	20 (30.3)	14 (21.2)	54 (81.8)	35 (53.0)*
	Absent	n = 453	356 (78.6)	91 (20.1)	63 (13.9)	313 (69.1)	**133 (29.4)**
Mental and psychological	Present	n = 129	112 (86.8)	38 (29.5)	26 (20.2)	103 (79.8)	62 (48.1)*
	Absent	n = 390	303 (77.7)	73 (18.7)	51 (13.1)	264 (67.7)	**106 (27.2)**

*Chi-square comparing the use of corresponding form of CAM between those with and without the given health problem was significant after Bonferroni adjustment for multiple comparisons (p-value < 0.0006).

**Based on females only, n = 290.

Health problems of urologic, kidney, endocrine, skin, cancer, chronic pain and ‘other’ nature are omitted from table due to the small number of individuals with the conditions (n < 50). No significant results were found in these conditions.

The reported use of dietary supplements, herbal medicines, and other ingestible products can be found in Tables 7 and 3. 167 of the respondents (32.2%) reported use of at least one herbal medicine or supplement during the past year, with 62 of these respondents (11.9%) using two or more products. The most common primary motivations for using dietary supplements, herbal medicines, and other ingestible products were for health maintenance (49.0%) and long-term illnesses (21.2%). Of those who reported use of these products, 74.6% thought these products were somewhat or very helpful, and only 27.7% reported their use of products to their physicians. Respondents with certain self-reported medical problems were significantly more likely to use CAM products than other study participants. These included those with musculoskeletal, gastrointestinal, allergic, and psychological illnesses (Table 6).

**Table 7:** **Number of different CAM products used by Individuals**

Number of CAM products used in past year	Number (%) of people*
1 or more	167 (32.2%)
2 or more	62 (11.9%)
3 or more	21 (4.0%)
4 or more	8 (1.5%)

*Denominator is total study respondent number of 519.

### Healthcare providers seen and CAM treatments from physicians

Table 4 displays the respondents’ visits to various healthcare providers. Overall, 111 respondents (21.4%) visited at least one alternative healthcare provider in the past year, 32 of whom visited two ore more providers. The alternative providers seen most frequently were massage therapists (n = 39, 7.5%), chiropractors (n = 35, 6.7%), acupuncturists (n = 24, 4.6%), and bonesetters (n = 21, 4.0%). Most participants’ primary motivation for seeing these providers was for long-term illnesses or health maintenance. For every alternative provider seen, at least 66.6% of respondents perceived their visits as very or somewhat helpful. Respondents with certain self-reported medical problems were more likely to see alternative providers than other study participants. These included those with musculoskeletal and neurologic problems.

The alternative treatments that respondents received from their primary care physicians can be found in Table 5. Overall, 77 respondents (14.8%) received some type of alternative therapy from their physician in the past year, with 68 respondents receiving one type of therapy, and 9 respondents receiving two types of therapy. Kampo (n = 47, 9.1%) was the most commonly prescribed treatment. Supplements (n = 18, 3.5%) and acupuncture (n = 12, 2.3%) were also occasionally prescribed. Most respondents reported that alternative treatments from physicians were prescribed for long-term illnesses. 82.1% of respondents who received treatments perceived these treatments as very or somewhat helpful.

## Discussion

These data illustrate widespread use of and positive attitudes about CAM in these rural Japanese primary care populations. They also reveal important differences regarding gender, education, and health status in relation to CAM use. Female respondents, respondents with higher levels of education, and respondents with poorer overall health statuses were more likely to use certain types of CAM than those without these characteristics. Specifically, respondents with musculoskeletal, neurologic, gastrointestinal, allergic, and psychological illnesses were more likely to use certain types of CAM than respondents without these illnesses.

These data delineate commonly used CAM treatments and practitioners in rural Japan. Specifically, about a third of respondents reported use of pain relief pads, massage by self or family, and dietary supplements/herbs. In addition, about 1 of 13 respondents are using massage therapists and chiropractors, and one in 11 participants reported receiving kampo from a primary care physician. This seemed surprisingly low to the authors since the research was conducted during the winter months, when kampo formulations for the common cold are often used.

Over three quarters of participants said that they did not report their use of CAM therapies to physicians. A nationwide oncology survey showed similar results, with 60.7% of CAM users indicating that they did not consult a physician about CAM use [20]. A large review article investigated this issue as well, and found that rates of non-disclosure of CAM use to physicians ranged from 12-77% in 12 selected studies from various countries. In this review, the top three reasons that respondents listed for not revealing their CAM use to medical providers were concerns about a negative response, beliefs that the practitioner did not need to know, and the fact that the practitioner did not ask [11]. The reasons for not disclosing to physicians in our study may be similar, and may also be that rural Japanese patients feel there is not enough time in their brief encounter to discuss their use of CAM. Regardless of the reasons, physicians in rural Japan would benefit from familiarizing themselves with the most common types of CAM modalities, and inquiring about CAM use during patient encounters.

### Comparison with previous studies

The use of different survey instruments, as well as varying definitions of CAM, make direct comparisons between studies on CAM consumption difficult. While the I-CAM-Q is a standardized survey that aims to address this issue, it has not yet been widely implemented in rural, urban, and nationwide studies on CAM use. Thus, caution is still needed when comparing these studies and interpreting results. Because most studies do not include prayer in their definition of CAM, we will use our prevalence number that excludes prayer of 77.6% when making comparisons.

The 77.6% prevalence of CAM use in this rural Japanese study exceeds that of CAM use in nation-wide population studies in England, Germany, Australia, and the USA, where the 12-month prevalence was reported to be 26.3%, 42.3%, 68.9%, and 38.3% respectively [5–8]. However, previous research has demonstrated that CAM use is generally more common in rural areas than urban areas, and thus it is important to examine our study in relation to rural-specific data to draw more appropriate cross-cultural comparisons [3].

The most robust data on CAM use in rural locations comes from North America and Australia, with major (n > 300) studies showing the prevalence of CAM use ranging from 40-70% [3]. This large range is likely the result of cultural differences between rural communities, as well as variations in study design and definitions of CAM. Still, the high prevalence of CAM use across numerous rural studies suggests that CAM modalities are commonly utilized in rural communities, which is consistent with our findings.

One methodologically similar study to ours in a northern Pennsylvania rural family medicine clinic showed a 47% prevalence of CAM use, with the most common modalities being chiropractic (17.2%), relaxation techniques (16.9%), herbal medicine (16.9%), and massage (14.2%). In that study, only 51% of respondents told their primary care physician about their CAM use [22]. Another similar study in a New South Wales community in rural Australia revealed that 70.3% of survey respondents had used one or more CAM modality in the past 12 months, with 62.7% having visited a CAM practitioner. Vitamin/mineral therapy was most common in that study (68.7%), followed by chiropractic (26.1%) and massage therapy (25.1%) [23]. Our study shows a higher prevalence of CAM use, with pain relief pads, dietary supplements/herbs, and massage therapy being most commonly used.

Our study also reveals that CAM use in this rural Japan sample is positively associated with certain characteristics, including being female, obtaining a higher level of education, and having a lower self-rated health status. Interestingly, this is not only directly in line with previous rural studies, but also with previous nation-wide cross-sectional studies in England, Germany, Australia, and the USA [3, 5–8]. This suggests a global theme, and that physicians in rural Japan – and perhaps worldwide – should be particularly alert to possible CAM use in patients with these characteristics.

While our study is the first to focus specifically on CAM use in rural Japan, there are many similarities between our results and the results of previous studies that focused on CAM use in different Japanese populations. Use of CAM by 77.6% of respondents is higher than findings from the survey performed in Tokyo primary care clinics [13], and very similar to the 76.0% using CAM in a nation-wide telephone study [21]. The reason for the higher prevalence than the Tokyo clinic study may be due to differences in survey instruments and study designs, may be due to specific practices by the doctors in the chosen clinics, or may be due to a higher prevalence of CAM use in rural Japanese primary care populations.

The use of massage by self or family in our study is slightly higher than the use of massage in previous Japanese studies: 21.4% of respondents reported using massage in the 2008 study in Tokyo, and 14.8% reported massage use in the 2002 nation-wide telephone survey [13, 21]. These data from Japan show a higher utilization of massage than in studies from England (13%) and the USA (11%), and similar to the use of massage from a nation-wide study in Australia (27%) [5, 7, 9].

Our results showing that 32.2% of respondents used some form of kampo, dietary supplements, or herbs in the past year are consistent with the previous research in Japan indicating the widespread use of these products. The 2008 Tokyo primary care research showed that 17.1% of respondents used vitamins, 11.3% used dietary supplements, and 9.3% used kampo [13]. The 2002 nation-wide telephone study showed 43.1% of respondents used nutritional drinks, 43.1% used dietary supplements, and 17.2% used herbs or over-the-counter kampo [21]. The high consumption of CAM products was also revealed by a nationwide survey of oncology patients, in which 44.6% of oncology patients used CAM, and 96.2% of CAM users used CAM products [20].

The study data regarding the utilization of alternative providers indicates that the most commonly visited healthcare providers other than physicians were massage therapists, chiropractors, and acupuncturists. Previous studies in Japan have demonstrated similar data regarding the use of chiropractors (7.1% and 6.5% in two studies) and acupuncturists (6.7% and 6.7% in two studies) [13, 21]. The specific use of massage therapists was not measured in either of the previous studies.

Interestingly, there were no significant differences in the utilization of alternative treatments received from physicians among the three clinic sites, suggesting that CAM treatments were spread out relatively equally amongst the different providers at each site. Several studies have demonstrated that kampo medicine is commonly prescribed by Japanese physicians, including a 1999 study in which 70% of Japanese physicians reported prescribing kampo in patient care [16]. Our study supports the notion that kampo is by far the most common type of CAM prescribed by physicians, as 9.1% of respondents reported receipt of kampo from physicians. All other forms of CAM investigated were rarely prescribed.

### The Japanese I-CAM-Q

On a methodological note, this is the first known study to use the I-CAM-Q in Japan. The consistency of the findings with previous studies using different surveys suggests the instrument’s psychometrics are robust. This version also identified CAM practices not investigated in the previous studies, such as the use of massage therapists and pain relief pads. The research team invested heavily in identifying modalities likely to be used in Japan, and this appears to have been successful. This underscores both the flexibility of the I-CAM-Q as a tool, and the value of adapting it for local practices.

### Limitations and bias considerations

While this research was conducted in three rural clinics, the degree these clinics are representative nationally is hard to discern. The consistency of these findings with previous studies suggests that the estimates of use are reasonable. However, given the relatively small sample size, some degree of sampling bias is likely present when extrapolating these results to all rural Japanese family medicine clinics. It is likely that there will be some local variations in CAM use depending on factors such as access to CAM practitioners, individual physician variation relative to kampo dispensing practices, and demographic differences between varying rural Japanese communities. As all three sites provide family medicine resident training, it is also likely that resident prescribing practices are influenced by their teachers.

Regarding information bias, some degree of recall bias is likely present, given that our study design asks participants to retroactively list CAM usage over the course of the past year. Additionally, for a predominantly elderly population, the survey was long, and some participants left items blank, especially towards the end of the survey. For example, 15.0% of participants did not complete the demographics section at the end of the survey. Moreover, from the interviewer’s perspective, it seemed that elderly visitors were more likely to decline the survey than younger visitors, adding an additional level of potential selection bias. Regardless, the results describe the motivations for use and helpfulness of various types of CAM for a substantial amount of patients and visitors in the three rural clinic sites.

## Conclusions

These data indicate that CAM modalities such as massage, dietary supplements, and kampo use among patients, and family members accompanying them to clinics, in rural Japan are common. These data are highly consistent with previous research in Japan using random telephone sampling and higher than the prevalence of 49.6% use in urban clinics. The results also indicate that pain relief pads are commonly used in this population, which has not been demonstrated in previous research. Being female, having higher education, and suffering from chronic health problems were predictors of higher likelihood of CAM use, which is highly consistent with previous research in rural communities and nation-wide studies from Australia, England, Germany, and the USA. Importantly, over three-quarters of CAM therapies used were not reported by CAM users to their primary care physicians. Given the high prevalence of CAM use in this population, we conclude that physicians in rural Japan would benefit by familiarizing themselves with the benefits and risks of commonly used CAM modalities, and may be able to provide better care by routinely inquiring about CAM use with patients. Finally, the findings elicited using the I-CAM-Q, and their consistency with previous research, suggests that the I-CAM-Q is a robust instrument for assessing CAM use.

## Electronic supplementary material

Additional file 1: **Official IRB Exemption from Review.** (DOCX 101 KB)

Additional file 2: **Adapted English Version of I-CAM-Q.** (DOCX 161 KB)

Additional file 3: **English Explanatory Face Sheet for I-CAM-Q.** (DOCX 107 KB)

Additional file 4: **English Demographics page for I-CAM-Q.** (DOCX 20 KB)

Additional file 5: **Adapted Japanese Version of I-CAM-Q.** (DOCX 155 KB)

Additional file 6: **Japanese Explanatory Face Sheet for I-CAM-Q.** (DOCX 98 KB)

Additional file 7: **Japanese Demographics page for I-CAM-Q.** (DOCX 21 KB)

## Abbreviations

CAM: Complementary and Alternative Medicine
I-CAM-Q: International Complementary and Alternative Medicine Questionnaire
NCCAM: National Center for Complementary and Alternative Medicine.
