# Supplementary material for: Complementary and alternative medicine use by visitors to rural Japanese family medicine clinics: results from the international complementary and alternative medicine survey
Source: BMC Complement Altern Med. 2014 Sep 25;14:360. doi: 10.1186/1472-6882-14-360 (PMC4192731; doi:10.1186/1472-6882-14-360)
Supplement: Supplementary file 1 — Additional file 1: Official IRB Exemption from Review. (DOCX 101 KB) [file 12906_2013_1938_MOESM1_ESM.docx]

**To:** Michael Fetters

**From:**

| \| Michael \| Geisser \| \| --- \| --- \| \| Alan \| Sugar \| |
| --- | --- | --- | --- | --- |

**Cc:**

| \| Ayaka \| Yajima \| \| --- \| --- \| \| Michael \| Fetters \| \| Gregory \| Shumer \| \| Satoko \| Motohara \| \| Sara \| Warber \| \| Judy \| Connelly \| |
| --- | --- | --- | --- | --- | --- | --- | --- | --- | --- | --- | --- | --- |

**Subject:** Notice of Exemption for [HUM00070306]

**SUBMISSION INFORMATION:**
Title: Complementary and alternative medicine use among primary care patients in rural Japan – Data Collection
Full Study Title (if applicable): Complementary and alternative medicine use among primary care patients in rural Japan – Data Collection
Study eResearch ID: [HUM00070306](https://eresearch.umich.edu/eresearch?PageID=HUM00070306) 
Date of this Notification from IRB: 12/3/2012 
Date of IRB Exempt Determination: 12/3/2012
UM Federalwide Assurance: FWA00004969 expiring on 6/13/2014 
OHRP IRB Registration Number(s):

**IRB EXEMPTION STATUS:**
The IRBMED has reviewed the study referenced above and determined that, as currently described, it is exempt from ongoing IRB review, per the following federal exemption category:

**EXEMPTION #2 of the 45 CFR 46.101.(b):**
Research involving the use of educational tests (cognitive, diagnostic, aptitude, achievement), survey procedures, interview procedures or observation of public behavior, unless: (i) information obtained is recorded in such a manner that human subjects can be identified, directly or through identifiers linked to the subjects; and (ii) any disclosure of the human subjects' responses outside the research could reasonably place the subjects at risk of criminal or civil liability or be damaging to the subjects' financial standing, employability, or reputation.

Note that the study is considered exempt as long as any changes to the use of human subjects (including their data) remain within the scope of the exemption category above. Any proposed changes that may exceed the scope of this category, or the approval conditions of any other non-IRB reviewing committees, must be submitted as an amendment through eResearch.

Although an exemption determination eliminates the need for ongoing IRB review and approval, you still have an obligation to understand and abide by generally accepted principles of responsible and ethical conduct of research. Examples of these principles can be found in the Belmont Report as well as in guidance from professional societies and scientific organizations.

**SUBMITTING AMENDMENTS VIA eRESEARCH:**
You can access the online forms for amendments in the eResearch workspace for this exempt study, referenced above.

**ACCESSING EXEMPT STUDIES IN eRESEARCH:**
Click the "Exempt and Not Regulated" tab in your eResearch home workspace to access this exempt study.

| 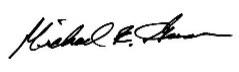 | 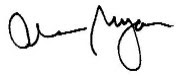 |
| --- | --- |
| **Michael Geisser** Co-chair, IRBMED | **Alan Sugar** Co-chair, IRBMED |
