# Supplementary material for: Complementary and alternative medicine use by visitors to rural Japanese family medicine clinics: results from the international complementary and alternative medicine survey
Source: BMC Complement Altern Med. 2014 Sep 25;14:360. doi: 10.1186/1472-6882-14-360 (PMC4192731; doi:10.1186/1472-6882-14-360)
Supplement: Supplementary file 2 — Additional file 2: Adapted English Version of I-CAM-Q. (DOCX 161 KB) [file 12906_2013_1938_MOESM2_ESM.docx]

**This questionnaire will ask you about treatments and therapies you have used**

**for your health problems or for health maintenance.**

**1. Visiting health care providers:** Health problems may be treated by

a variety of medical care, complementary and alternative medicine, and folk remedies

| Have you seen any of the following providers in the **past year**? Please circle “yes” or “no” | | Number of times you saw this provider in the **last** **3 months**? | Please choose **one** of the following (1-4) that best describes the **main reason** you saw this provider.  　1 For an acute illness/condition that lasted less  than one month  　2 For a long-term illness/condition that lasted  more than one month  　3 Health maintenance  　4 Other (Please specify the other reason) | How helpful was it for you to see this provider?  　1 Very helpful  　2 Somewhat helpful  　3 Not helpful  　4 Don’t know | Did you tell your primary care doctor that you saw this provider? |
| --- | --- | --- | --- | --- | --- |
| Physician | Yes　No | ＿＿times | 1　 2　 3　 4　 Reason：＿＿＿＿＿＿＿ | 1　　 2　　 3　　 4 |  |
| Rehabilitation Therapist | Yes　No | ＿＿times | 1　 2　 3　 4　 Reason：＿＿＿＿＿＿＿ | 1　　 2　　 3　　 4 | Yes　No |
| Bonesetter | Yes　No | ＿＿times | 1　 2　 3　 4　 Reason：＿＿＿＿＿＿＿ | 1　　 2　　 3　　 4 | Yes　No |
| Chiropractor | Yes　No | ＿＿times | 1　 2　 3　 4　 Reason：＿＿＿＿＿＿＿ | 1　　 2　　 3　　 4 | Yes　No |
| Massage Therapist | Yes　No | ＿＿times | 1　 2　 3　 4　 Reason：＿＿＿＿＿＿＿ | 1　　 2　　 3　　 4 | Yes　No |
| Accupuncturist/Moxa-Cauterizer | Yes　No | ＿＿times | 1　 2　 3　 4　 Reason：＿＿＿＿＿＿＿ | 1　　 2　　 3　　 4 | Yes　No |
| Kampo Practitioner | Yes　No | ＿＿times | 1　 2　 3　 4　 Reason：＿＿＿＿＿＿＿ | 1　　 2　　 3　　 4 | Yes　No |
| Qigong Therapist | Yes　No | ＿＿times | 1　 2　 3　 4　 Reason：＿＿＿＿＿＿＿ | 1　　 2　　 3　　 4 | Yes　No |
| Spiritual healer | Yes　No | ＿＿times | 1　 2　 3　 4　 Reason：＿＿＿＿＿＿＿ | 1　　 2　　 3　　 4 | Yes　No |
| Other:  ＿＿＿＿＿＿ | Yes　No | ＿＿times | 1　 2　 3　 4　 Reason：＿＿＿＿＿＿＿ | 1　　 2　　 3　　 4 | Yes　No |

**2. Alternative treatments received from physicians:** Some physicians may provide alternative treatments listed below.

***If you have not seen a physician in the past year, please go to question 3.**

| Have you received any of the following treatments or advice from a **physician** in the **past year**? | | Number of times you received this treat-ment in the **last 3 months**? | Please choose **one** of the following (1-4) that best describes the **main reason** you last received this treatment.  　1 For an acute illness/condition that lasted less  than one month  　2 For a long-term illness/condition that lasted  more than one month  　3 Health maintenance  　4 Other (Please specify the other reason) | How helpful was it to receive this treatment?  　1 Very helpful  　2 Somewhat helpful  　3 Not helpful  　4 Don’t know | Did you tell your primary care doctor that you received this treatment? |
| --- | --- | --- | --- | --- | --- |
| Kampo | Yes　No | ＿＿times | 1　 2　 3　 4　 Reason：＿＿＿＿＿＿＿ | 1　　 2　　 3　　 4 | Yes　No |
| Supplements | Yes　No | ＿＿times | 1　 2　 3　 4　 Reason：＿＿＿＿＿＿＿ | 1　　 2　　 3　　 4 | Yes　No |
| Acupuncture and moxibustion | Yes　No | ＿＿times | 1　 2　 3　 4　 Reason：＿＿＿＿＿＿＿ | 1　　 2　　 3　　 4 | Yes　No |
| Qigong | Yes　No | ＿＿times | 1　 2　 3　 4　 Reason：＿＿＿＿＿＿＿ | 1　　 2　　 3　　 4 | Yes　No |
| Other:  ＿＿＿＿＿＿ | Yes　No | ＿＿times | 1　 2　 3　 4　 Reason：＿＿＿＿＿＿＿ | 1　　 2　　 3　　 4 | Yes　No |

**3. Self Help Practices:** This section will ask you about self-help practices or folk remedies you have used when you had health problems or to maintain health.

| Have you used any of the following **self-help** practices in the **past year**? | | Number of times you used this practice in the **last 3 months**? | Please choose **one** of the following (1-4) that best describes the **main reason** you last used this practice.  　1 For an acute illness/condition that lasted less  than one month  　2 For a long-term illness/condition that lasted  more than one month  　3 Health maintenance  4 Other (Please specify the other reason) | How helpful did you find this practice?  　1 Very helpful  　2 Somewhat helpful  　3 Not helpful  　4 Don’t know | Did you tell your primary care doctor that you used this practice/ therapy? |
| --- | --- | --- | --- | --- | --- |
| Zen/Meditation | Yes　No | ＿＿times | 1　 2　 3　 4　 Reason：＿＿＿＿＿＿＿ | 1　　 2　　 3　　 4 | Yes　No |
| Tai Chi/Qigong | Yes　No | ＿＿times | 1　 2　 3　 4　 Reason：＿＿＿＿＿＿＿ | 1　　 2　　 3　　 4 | Yes　No |
| Yoga | Yes　No | ＿＿times | 1　 2　 3　 4　 Reason：＿＿＿＿＿＿＿ | 1　　 2　　 3　　 4 | Yes　No |
| Cupping | Yes　No | ＿＿times | 1　 2　 3　 4　 Reason：＿＿＿＿＿＿＿ | 1　　 2　　 3　　 4 | Yes　No |
| Moxibustion (Moxa) | Yes　No | ＿＿times | 1　 2　 3　 4　 Reason：＿＿＿＿＿＿＿ | 1　　 2　　 3　　 4 | Yes　No |
| Pain relief pads | Yes　No | ＿＿times | 1　 2　 3　 4　 Reason：＿＿＿＿＿＿＿ | 1　　 2　　 3　　 4 | Yes　No |
| Hot-spring Therapy | Yes　No | ＿＿times | 1　 2　 3　 4　 Reason：＿＿＿＿＿＿＿ | 1　　 2　　 3　　 4 | Yes　No |
| Massage done by self or family members | Yes　No | ＿＿times | 1　 2　 3　 4　 Reason：＿＿＿＿＿＿＿ | 1　　 2　　 3　　 4 | Yes　No |
| Massage Device | Yes　No | ＿＿times | 1　 2　 3　 4　 Reason：＿＿＿＿＿＿＿ | 1　　 2　　 3　　 4 | Yes　No |
| Electrotherapy Device  (Not massage) | Yes　No | ＿＿times | 1　 2　 3　 4　 Reason：＿＿＿＿＿＿＿ | 1　　 2　　 3　　 4 | Yes　No |
| Have you used any of the following **self-help** practices in the **past year**? | | Number of times you used this practice in the **last 3 months**? | Please choose **one** of the following (1-4) that best describes the **main reason** you last used this practice.  　1 For an acute illness/condition that lasted less  than one month  　2 For a long-term illness/condition that lasted  more than one month  　3 Health maintenance  4 Other (Please specify the other reason) | How helpful did you find this practice?  　1 Very helpful  　2 Somewhat helpful  　3 Not helpful  　4 Don’t know | Did you tell your primary care doctor that you used this practice/ therapy? |
| Praying for own health | Yes　No | ＿＿times | 1　 2　 3　 4　 Reason：＿＿＿＿＿＿＿ | 1　　 2　　 3　　 4 | Yes　No |
| Attending traditional healing ceremony (temples, shrines, etc) for health recovery/ promotion | Yes　No | ＿＿times | 1　 2　 3　 4　 Reason：＿＿＿＿＿＿＿ | 1　　 2　　 3　　 4 | Yes　No |
| Wearing talisman for health recovery/ promotion | Yes　No | ＿＿times | 1　 2　 3　 4　 Reason：＿＿＿＿＿＿＿ | 1　　 2　　 3　　 4 | Yes　No |
| Aromatherapy | Yes　No | ＿＿times | 1　 2　 3　 4　 Reason：＿＿＿＿＿＿＿ | 1　　 2　　 3　　 4 | Yes　No |
| Dietary Therapy (For illnesses and allergies) | Yes　No | ＿＿times | 1　 2　 3　 4　 Reason：＿＿＿＿＿＿＿ | 1　　 2　　 3　　 4 | Yes　No |
| Other:  ＿＿＿＿＿＿＿＿ | Yes　No | ＿＿times | 1　 2　 3　 4　 Reason：＿＿＿＿＿＿＿ | 1　　 2　　 3　　 4 | Yes　No |

**4. Kampo, Herbal Medicine, Herb, Supplements, etc:** This section will ask you about tablets, capsules, liquids, creams, and other products you have used.

| Have you used any of the following products in the **past year**?   - **Kampo** - **Herbal medicine（or its infusion）** - **Herb** - **Supplement** - **Vitamin** - **Energy drink, etc**   If so, please **specify** below. | Do you **currently** use this product? | Please choose **one** of the following (1-4) that best describes the **main reason** you last used this product.  　1 For an acute illness/condition that lasted less  than one month  　2 For a long-term illness/condition that lasted  more than one month  　3 Health maintenance  　4 Other (Please specify the other reason) | How helpful did you find this product?  　1 Very helpful  　2 Somewhat helpful  　3 Not helpful  　4 Don’t know | Did you tell your primary care doctor that you used this product? |
| --- | --- | --- | --- | --- |
| Example: Kakkonto | Yes　No | 1　 2　 3　 4　 Reason：＿＿＿＿＿＿＿ | 1　　 2　　 3　　 4 | Yes　No |
| Example：Goma-Sesamin | Yes　No | 1　 2　 3　 4　 Reason：＿＿＿＿＿＿＿ | 1　　 2　　 3　　 4 | Yes　No |
| ＿＿＿＿＿＿＿＿____ | Yes　No | 1　 2　 3　 4　 Reason：＿＿＿＿＿＿＿ | 1　　 2　　 3　　 4 | Yes　No |
| ＿＿＿＿＿＿＿＿____ | Yes　No | 1　 2　 3　 4　 Reason：＿＿＿＿＿＿＿ | 1　　 2　　 3　　 4 | Yes　No |
| ＿＿＿＿＿＿＿＿____ | Yes　No | 1　 2　 3　 4　 Reason：＿＿＿＿＿＿＿ | 1　　 2　　 3　　 4 | Yes　No |
| ＿＿＿＿＿＿＿＿____ | Yes　No | 1　 2　 3　 4　 Reason：＿＿＿＿＿＿＿ | 1　　 2　　 3　　 4 | Yes　No |
| ＿＿＿＿＿＿＿＿____ | Yes　No | 1　 2　 3　 4　 Reason：＿＿＿＿＿＿＿ | 1　　 2　　 3　　 4 | Yes　No |
| ＿＿＿＿＿＿＿＿____ | Yes　No | 1　 2　 3　 4　 Reason：＿＿＿＿＿＿＿ | 1　　 2　　 3　　 4 | Yes　No |
| ＿＿＿＿＿＿＿＿____ | Yes　No | 1　 2　 3　 4　 Reason：＿＿＿＿＿＿＿ | 1　　 2　　 3　　 4 | Yes　No |
| ＿＿＿＿＿＿＿＿____ | Yes　No | 1　 2　 3　 4　 Reason：＿＿＿＿＿＿＿ | 1　　 2　　 3　　 4 | Yes　No |

**Thank you for your participation!**
