# Supplementary material for: Complementary and alternative medicine use by visitors to rural Japanese family medicine clinics: results from the international complementary and alternative medicine survey
Source: BMC Complement Altern Med. 2014 Sep 25;14:360. doi: 10.1186/1472-6882-14-360 (PMC4192731; doi:10.1186/1472-6882-14-360)
Supplement: Supplementary file 3 — Additional file 3: English Explanatory Face Sheet for I-CAM-Q. (DOCX 107 KB) [file 12906_2013_1938_MOESM3_ESM.docx]

**Your cooperation in a study regarding**

**Complementary and Alternative Medicine and Folk remedy use**

Greetings,

We would like to invite you to take part in a research study called “Complementary and Alternative Medicine and Folk remedy use in Smaller Cities in Japan.”

Researchers from the University of Michigan and Georgetown University are working together on this study, with the support from Shizuoka Family Medicine Program and Yuge Family Medicine Clinic.

This questionnaire will ask you about treatments and therapies you use for your health problems or for health maintenance.

Participation in this study is voluntary. Your decision will not affect the treatments you receive. Completion of the survey usually takes about 5-10 minutes.

Your answers will be anonymous. This means that nobody will be able to link your name to your answers. The information gathered will be securely saved until the completion of the analysis.

If you are willing to take part in our research, please complete the attached survey now and return it to the specified collection box. If you do not want to take the survey, please return it to the person who handed it to you without answering the questions.

Thank you in advance for your consideration.

Research Team

**If you have any questions, please feel free to contact any of the following places in Japanese.**

Principal Investigator:

Michael D. Fetters, MD, MPH, MA

University of Michigan Department of Family Medicine/Shizuoka Family Medicine Residency Training Program Advisor

1018 Fuller St. Ann Arbor, MI 48104‐1213 USA

Phone: 001-734‐998‐7120 x341

Email: mfetters@umich.edu

Supported by:

Shizuoka Family Medicine Program (Kikugawa Family Medicine Center)

1055-1 Akatsuchi, Kikugawa, Shizuoka 437-1507

Phone: 0537-73-5551 
Email: [sfm@tenor.ocn.ne.jp](mailto:sfm@tenor.ocn.ne.jp?subject=%E3%81%8A%E5%95%8F%E3%81%84%E5%90%88%E3%82%8F%E3%81%9B)

<http://www.shizuoka-fm.org/>

Yuge Family Medicine Clinic

1825 Yuge, Gamogun Ryuocho, Shiga 520-2501

Phone: 0748-57-1141

http://yugemed.com/

University of Michigan ‐Medical School Institutional Review Board (IRBMED)

Research project number: HUM00069566

2800 Plymouth Road, Bldg 200, Rm 2086 Ann Arbor, MI 48109‐2800 USA

Phone: 001‐734‐763‐4768
