# Supplementary material for: Complementary and alternative medicine use by visitors to rural Japanese family medicine clinics: results from the international complementary and alternative medicine survey
Source: BMC Complement Altern Med. 2014 Sep 25;14:360. doi: 10.1186/1472-6882-14-360 (PMC4192731; doi:10.1186/1472-6882-14-360)
Supplement: Supplementary file 4 — Additional file 4: English Demographics page for I-CAM-Q. (DOCX 20 KB) [file 12906_2013_1938_MOESM4_ESM.docx]

**Your age: ____________________ years old**

**Your gender:**

1 Male

2 Female

**The highest level of education you have completed:**

1 Middle School or the equivalent

2 Some high school or equivalent

3 High school graduate or equivalent

4 Some college or equivalent

5 Two year college or technical school graduate or equivalent

6 Four year college graduate or equivalent

7 More studies after college or equivalent

**How would you rate your overall health?**

1 Excellent

2 Good

3 Fair

4 Poor

**Did you have any of the following health problems in the past year? Please circle all that apply.**

1 Musculoskeletal problem (Arthritis, muscle pain, neck stiffness, etc)

2 Cardiovascular problem (High blood pressure, heart disease, arrhythmia, etc)

3 Pulmonary problem (Pneumonia, asthma, common cold, etc)

4 Neurologic problem (Numbness and tingling, autonomic imbalance, etc)

5 Gastrointestinal problem (Heartburn, stomachache, constipation)

6 Gynecologic problem (Menstrual irregularity, menstrual pain, etc)

7 Urologic problem (Bladder infection, frequent urination, prostatic hypertrophy, etc)

8 Kidney problem (Kidney stone, kidney failure, etc)

9 Endocrine problem (Thyroid, diabetes, etc)

10 Skin problem (Burn, dermatitis, eczema, etc)

11 Cancer

12 Allergies

13 Chronic pain

14 Mental and Psychological problem (Malaise, stress, insomnia, depression, etc)

15 Other ___________________________________________
