# Supplementary material for: Complementary and alternative medicine use by visitors to rural Japanese family medicine clinics: results from the international complementary and alternative medicine survey
Source: BMC Complement Altern Med. 2014 Sep 25;14:360. doi: 10.1186/1472-6882-14-360 (PMC4192731; doi:10.1186/1472-6882-14-360)
Supplement: Supplementary file 5 — Additional file 5: Adapted Japanese Version of I-CAM-Q. (DOCX 155 KB) [file 12906_2013_1938_MOESM5_ESM.docx]

**このアンケートは、あなたが心身に不調がある時もしくは健康維持のために、　　　　　　　　　　　　　　　　　　　　　　　　　　　　　どのような治療法や健康法を用いたかについておたずねするものです。**

**1. 専門家への相談:**心身の不調は、様々な医療、代替医療、民間療法で治療することができます。

| **過去１年以内**に以下の専門家に相談しましたか？「はい」　　　　　「いいえ」に○をしてください。 | | **過去３ヶ月以内**に　何回この専門家に相談しましたか？ | この専門家に相談した***主な理由***は何ですか？１〜４の中で最もよくあてはまるものを***一つ***選んでください。  　１ 一ヶ月以内に治った急性の病気や不調  　２ 一ヶ月以上治らなかった慢性的な病気や不調  　３ 健康維持  　４ その他（理由を具体的に記入してください） | この専門家に相談したことは　どのくらい役に立ちましたか？  　１ とても役立った  　２ ある程度役立った  　３ 役に立たなかった  　４ わからない | あなたのかかりつけ医に、この専門家に相談した　ことを話しましたか？ |
| --- | --- | --- | --- | --- | --- |
| 医師 | はい　いいえ | ＿＿＿回 | １　 ２　 ３　 ４　 理由：＿＿＿＿＿＿＿＿ | １　　 ２　　 ３　　 ４ |  |
| リハビリ療法士 | はい　いいえ | ＿＿＿回 | １　 ２　 ３　 ４　 理由：＿＿＿＿＿＿＿＿ | １　　 ２　　 ３　　 ４ | はい　いいえ |
| 接骨師 | はい　いいえ | ＿＿＿回 | １　 ２　 ３　 ４　 理由：＿＿＿＿＿＿＿＿ | １　　 ２　　 ３　　 ４ | はい　いいえ |
| 整体師・  カイロプラクター | はい　いいえ | ＿＿＿回 | １　 ２　 ３　 ４　 理由：＿＿＿＿＿＿＿＿ | １　　 ２　　 ３　　 ４ | はい　いいえ |
| あんま・指圧・  マッサージ師 | はい　いいえ | ＿＿＿回 | １　 ２　 ３　 ４　 理由：＿＿＿＿＿＿＿＿ | １　　 ２　　 ３　　 ４ | はい　いいえ |
| 鍼灸師  （はり・きゅう） | はい　いいえ | ＿＿＿回 | １　 ２　 ３　 ４　 理由：＿＿＿＿＿＿＿＿ | １　　 ２　　 ３　　 ４ | はい　いいえ |
| 漢方医 | はい　いいえ | ＿＿＿回 | １　 ２　 ３　 ４　 理由：＿＿＿＿＿＿＿＿ | １　　 ２　　 ３　　 ４ | はい　いいえ |
| 気功師 | はい　いいえ | ＿＿＿回 | １　 ２　 ３　 ４　 理由：＿＿＿＿＿＿＿＿ | １　　 ２　　 ３　　 ４ | はい　いいえ |
| スピリチュアル　ヒーラー・霊能者 | はい　いいえ | ＿＿＿回 | １　 ２　 ３　 ４　 理由：＿＿＿＿＿＿＿＿ | １　　 ２　　 ３　　 ４ | はい　いいえ |
| その他：  ＿＿＿＿＿＿ | はい　いいえ | ＿＿＿回 | １　 ２　 ３　 ４　 理由：＿＿＿＿＿＿＿＿ | １　　 ２　　 ３　　 ４ | はい　いいえ |

**2. 医師から受けた代替医療：**医師が以下のような代替医療を行う場合もあります。

**＊過去１年以内に医師に受診しなかった場合、次のページ進んでください。**

| **過去１年以内**に**医師から**以下の治療またはアドバイスを受けましたか？ | | **過去３ヶ月以内**に何回この治療を受けましたか？ | 最も最近この治療を受けた***主な理由***は何ですか？１〜４の中で最もよくあてはまるものを***一つ***選んでください。  　１ 一ヶ月以内に治った急性の病気や不調  　２ 一ヶ月以上治らなかった慢性的な病気や不調  　３ 健康維持  　４ その他（理由を具体的に記入してください） | この治療を受けたことはどのくらい役に立ちましたか？  　１ とても役立った  　２ ある程度役立った  　３ 役に立たなかった  　４ わからない | あなたのかかりつけ医に、この治療を　受けたことを話しましたか？ |
| --- | --- | --- | --- | --- | --- |
| 漢方 | はい　いいえ | ＿＿＿回 | １　 ２　 ３　 ４　 理由：＿＿＿＿＿＿＿＿ | １　　 ２　　 ３　　 ４ | はい　いいえ |
| サプリメント | はい　いいえ | ＿＿＿回 | １　 ２　 ３　 ４　 理由：＿＿＿＿＿＿＿＿ | １　　 ２　　 ３　　 ４ | はい　いいえ |
| 鍼灸  （はり・きゅう） | はい　いいえ | ＿＿＿回 | １　 ２　 ３　 ４　 理由：＿＿＿＿＿＿＿＿ | １　　 ２　　 ３　　 ４ | はい　いいえ |
| 気功 | はい　いいえ | ＿＿＿回 | １　 ２　 ３　 ４　 理由：＿＿＿＿＿＿＿＿ | １　　 ２　　 ３　　 ４ | はい　いいえ |
| その他：  ＿＿＿＿＿＿ | はい　いいえ | ＿＿＿回 | １　 ２　 ３　 ４　 理由：＿＿＿＿＿＿＿＿ | １　　 ２　　 ３　　 ４ | はい　いいえ |

**3. 自分で行ったこと：**　あなたが心身に不調がある時もしくは健康維持のために用いた　　　　　　　　　　　　　　　　　　　　　　　　　自己治療や自分で行った民間療法についておたずねします。

| **過去１年以内**に以下のことを　　　**自分で**行いましたか？ | | **過去３ヶ月以内**に何回　この方法を行いましたか？ | 最も最近この方法を行った***主な理由***は何ですか？１〜４の中で、最もよくあてはまるものを***一つ***選んでください。  　１ 一ヶ月以内に治った急性の病気や不調  　２ 一ヶ月以上治らなかった慢性的な病気や不調  　３ 健康維持  　４ その他（理由を具体的に記入してください） | この方法はどのくらい役に立ちましたか？  　１ とても役立った  　２ ある程度役立った  　３ 役に立たなかった  　４ わからない | あなたのかかりつけ医に、この方法・　療法を行ったことを話しましたか？ |
| --- | --- | --- | --- | --- | --- |
| 禅・瞑想 | はい　いいえ | ＿＿＿回 | １　 ２　 ３　 ４　 理由：＿＿＿＿＿＿＿＿ | １　　 ２　　 ３　　 ４ | はい　いいえ |
| 太極拳・気功 | はい　いいえ | ＿＿＿回 | １　 ２　 ３　 ４　 理由：＿＿＿＿＿＿＿＿ | １　　 ２　　 ３　　 ４ | はい　いいえ |
| ヨガ | はい　いいえ | ＿＿＿回 | １　 ２　 ３　 ４　 理由：＿＿＿＿＿＿＿＿ | １　　 ２　　 ３　　 ４ | はい　いいえ |
| 吸い玉療法  （カッピング） | はい　いいえ | ＿＿＿回 | １　 ２　 ３　 ４　 理由：＿＿＿＿＿＿＿＿ | １　　 ２　　 ３　　 ４ | はい　いいえ |
| お灸（モグサ） | はい　いいえ | ＿＿＿回 | １　 ２　 ３　 ４　 理由：＿＿＿＿＿＿＿＿ | １　　 ２　　 ３　　 ４ | はい　いいえ |
| 湿布（しっぷ）薬 | はい　いいえ | ＿＿＿回 | １　 ２　 ３　 ４　 理由：＿＿＿＿＿＿＿＿ | １　　 ２　　 ３　　 ４ | はい　いいえ |
| 湯治（温泉療法） | はい　いいえ | ＿＿＿回 | １　 ２　 ３　 ４　 理由：＿＿＿＿＿＿＿＿ | １　　 ２　　 ３　　 ４ | はい　いいえ |
| 自分でまたは家族に　　指圧・マッサージを　　　　してもらった | はい　いいえ | ＿＿＿回 | １　 ２　 ３　 ４　 理由：＿＿＿＿＿＿＿＿ | １　　 ２　　 ３　　 ４ | はい　いいえ |
| マッサージ機 | はい　いいえ | ＿＿＿回 | １　 ２　 ３　 ４　 理由：＿＿＿＿＿＿＿＿ | １　　 ２　　 ３　　 ４ | はい　いいえ |
| 低周波・電気治療器  （マッサージを除く） | はい　いいえ | ＿＿＿回 | １　 ２　 ３　 ４　 理由：＿＿＿＿＿＿＿＿ | １　　 ２　　 ３　　 ４ | はい　いいえ |
| **過去１年以内**に以下のことを　　　 **自分で**行いましたか？ | | **過去３ヶ月以内**に何回　この方法を行いましたか？ | 最も最近この方法を行った***主な理由***は何ですか？１〜４の中で、最もよくあてはまるものを***一つ***選んでください。  　１ 一ヶ月以内に治った急性の病気や不調  　２ 一ヶ月以上治らなかった慢性的な病気や不調  　３ 健康維持  　４ その他（理由を具体的に記入してください） | この方法はどのくらい役に立ちましたか？  　１ とても役立った  　２ ある程度役立った  　３ 役に立たなかった  　４ わからない | あなたのかかりつけ医に、この方法・　療法を行ったことを話しましたか？ |
| 病気回復や  健康のために　　　　自分で拝んだ、祈った | はい　いいえ | ＿＿＿回 | １　 ２　 ３　 ４　 理由：＿＿＿＿＿＿＿＿ | １　　 ２　　 ３　　 ４ | はい　いいえ |
| 病気回復や健康の　ために寺社などを　　参拝した、または　　祈祷してもらった | はい　いいえ | ＿＿＿回 | １　 ２　 ３　 ４　 理由：＿＿＿＿＿＿＿＿ | １　　 ２　　 ３　　 ４ | はい　いいえ |
| 病気回復や健康のためお守りを持った | はい　いいえ | ＿＿＿回 | １　 ２　 ３　 ４　 理由：＿＿＿＿＿＿＿＿ | １　　 ２　　 ３　　 ４ | はい　いいえ |
| アロマセラピー | はい　いいえ | ＿＿＿回 | １　 ２　 ３　 ４　 理由：＿＿＿＿＿＿＿＿ | １　　 ２　　 ３　　 ４ | はい　いいえ |
| 食事療法（病気や　　　　　　アレルギーのため） | はい　いいえ | ＿＿＿回 | １　 ２　 ３　 ４　 理由：＿＿＿＿＿＿＿＿ | １　　 ２　　 ３　　 ４ | はい　いいえ |
| その他：  ＿＿＿＿＿＿＿＿ | はい　いいえ | ＿＿＿回 | １　 ２　 ３　 ４　 理由：＿＿＿＿＿＿＿＿ | １　　 ２　　 ３　　 ４ | はい　いいえ |

**4. 漢方薬・生薬・ハーブ・サプリメントなど：**あなたが用いた錠剤、カプセル、液体、塗り薬などについておたずねします。

| あなたが**過去１年以内**で最もよく使った   - **漢方薬** - **生薬（煎じ薬）** - **ハーブ** - **サプリメント** - **ビタミン剤** - **栄養ドリンク剤 など**   を**具体的に**下欄に記入してください。 | **現在**この薬　またはハーブを使っていますか？ | 最も最近この薬、ハーブまたはサプリメントを使った***主な理由***は何ですか？１〜４の中で、最もよくあてはまるものを***一つ***選んでください。  　１ 一ヶ月以内に治った急性の病気や不調  　２ 一ヶ月以上治らなかった慢性的な病気や不調  　３ 健康維持  　４ その他（理由を具体的に記入してください） | この薬、ハーブまたはサプリメントはどのくらい役に立ちましたか？  　１ とても役立った  　２ ある程度役立った  　３ 役に立たなかった  　４ わからない | あなたのかかりつけ医に、この薬、ハーブまたはサプリメントを使ったことを話しましたか？ |
| --- | --- | --- | --- | --- |
| ____**例：**葛根湯____ | はい　いいえ | １　 ２　 ３　 ４　 理由：＿＿＿＿＿＿＿＿ | １　　 ２　　 ３　　 ４ | はい　いいえ |
| **例：**ごまセサミン | はい　いいえ | １　 ２　 ３　 ４　 理由：＿＿＿＿＿＿＿＿ | １　　 ２　　 ３　　 ４ | はい　いいえ |
| ＿＿＿＿＿＿＿＿____ | はい　いいえ | １　 ２　 ３　 ４　 理由：＿＿＿＿＿＿＿＿ | １　　 ２　　 ３　　 ４ | はい　いいえ |
| ＿＿＿＿＿＿＿＿____ | はい　いいえ | １　 ２　 ３　 ４　 理由：＿＿＿＿＿＿＿＿ | １　　 ２　　 ３　　 ４ | はい　いいえ |
| ＿＿＿＿＿＿＿＿____ | はい　いいえ | １　 ２　 ３　 ４　 理由：＿＿＿＿＿＿＿＿ | １　　 ２　　 ３　　 ４ | はい　いいえ |
| ＿＿＿＿＿＿＿＿____ | はい　いいえ | １　 ２　 ３　 ４　 理由：＿＿＿＿＿＿＿＿ | １　　 ２　　 ３　　 ４ | はい　いいえ |
| ＿＿＿＿＿＿＿＿____ | はい　いいえ | １　 ２　 ３　 ４　 理由：＿＿＿＿＿＿＿＿ | １　　 ２　　 ３　　 ４ | はい　いいえ |
| ＿＿＿＿＿＿＿＿____ | はい　いいえ | １　 ２　 ３　 ４　 理由：＿＿＿＿＿＿＿＿ | １　　 ２　　 ３　　 ４ | はい　いいえ |
| ＿＿＿＿＿＿＿＿____ | はい　いいえ | １　 ２　 ３　 ４　 理由：＿＿＿＿＿＿＿＿ | １　　 ２　　 ３　　 ４ | はい　いいえ |
| ＿＿＿＿＿＿＿＿____ | はい　いいえ | １　 ２　 ３　 ４　 理由：＿＿＿＿＿＿＿＿ | １　　 ２　　 ３　　 ４ | はい　いいえ |

**ご協力ありがとうございました！**
