# Supplementary material for: Complementary and alternative medicine use by visitors to rural Japanese family medicine clinics: results from the international complementary and alternative medicine survey
Source: BMC Complement Altern Med. 2014 Sep 25;14:360. doi: 10.1186/1472-6882-14-360 (PMC4192731; doi:10.1186/1472-6882-14-360)
Supplement: Supplementary file 6 — Additional file 6: Japanese Explanatory Face Sheet for I-CAM-Q. (DOCX 98 KB) [file 12906_2013_1938_MOESM6_ESM.docx]

代替医療・民間療法の利用についての

アンケート調査ご協力のお願い

患者様各位

　皆様におかれましては、ますますご清栄のこととお喜び申し上げます。

　この度は、「日本の地方の小都市における代替医療・民間療法の利用についての研究」のアンケート調査へのご協力をお願いいたしたく存じます。

　このアンケートでは、心身に不調がある時や健康維持のために、皆さんがどのような治療法や健康法を使っていらっしゃるかをおたずねします。

　このアンケートへの参加は自由です。アンケートへの参加の有無やその内容によって患者様が不利益を被ることは一切ありません。回答には約５〜１０分ほどかかります。

　このアンケートは匿名ですので、あなたのお名前と回答が結びつくことはありません。いただいた回答はデータ分析が終わるまで安全な場所に保管されます。

　本研究は、静岡家庭医養成プログラムおよび弓削メディカルクリニックの後援のもと、米国ミシガン大学およびジョージタウン大学の研究者が行っています。

　私どもの研究にご協力いただける場合、アンケートにお答えいただき、調査票収集箱にご提出ください。アンケートに答えたくない場合、何も記入せず、研究スタッフにお返しください。

　お忙しい中恐れ入りますが、私どもの研究にご協力いただきますよう、よろしくお願い申し上げます。

研究チーム一同

**ご質問がある場合は、下記まで日本語でご連絡ください。**

主任研究者：

マイク　フェターズ　MD, MPH, MA

ミシガン大学家庭医療学科／静岡家庭医養成プログラム顧問

1018 Fuller St. Ann Arbor, MI 48104‐1213 USA

Phone （電話）: 001-734‐998‐7120 x341

Email （Eメール): mfetters@umich.edu

後援：

静岡家庭医養成プログラム（菊川市家庭医療センター内）

〒437-1507　静岡県菊川市赤土1055－１

電話：0537-73-5551 
Eメール：[sfm@tenor.ocn.ne.jp](mailto:sfm@tenor.ocn.ne.jp?subject=%E3%81%8A%E5%95%8F%E3%81%84%E5%90%88%E3%82%8F%E3%81%9B)

<http://www.shizuoka-fm.org/>

弓削メディカルクリニック

〒520-2501 滋賀県蒲生郡竜王町弓削1825

電話：0748-57-1141

http://yugemed.com/

ミシガン大学医学部倫理委員会

研究プロジェクト番号：HUM00069566

2800 Plymouth Road, Bldg 200, Rm 2086 Ann Arbor, MI 48109‐2800 USA

Phone（電話）: 001‐734‐763‐4768
