# Supplementary material for: Complementary and alternative medicine use by visitors to rural Japanese family medicine clinics: results from the international complementary and alternative medicine survey
Source: BMC Complement Altern Med. 2014 Sep 25;14:360. doi: 10.1186/1472-6882-14-360 (PMC4192731; doi:10.1186/1472-6882-14-360)
Supplement: Supplementary file 7 — Additional file 7: Japanese Demographics page for I-CAM-Q. (DOCX 21 KB) [file 12906_2013_1938_MOESM7_ESM.docx]

**あなたの年齢： _____________才**

**あなたの性別:**

1 男性

2 女性

**あなたの最終学歴:**

1 中学校卒業

2 高校中退

3 高校卒業

4 大学在籍中または大学中退

5 短期大学または専門学校卒業

6 四年制大学卒業

7 大学院など大学以上の教育

**ご自身の健康状態をどのように評価しますか？**

1 非常によい

2 よい

3 まあまあ

4 よくない

**あなたは過去一年以内に、以下のような病気や不調がありましたか？　　　　　　　　　　　当てはまるもの全てに○をしてください。**

1 筋骨格系の問題（関節炎、筋肉痛、肩こりなど）

2 心臓血管系の問題（高血圧、心臓病、不整脈など）

3 呼吸器系の問題（肺炎、喘息、風邪など）

4 神経系の問題（しびれ、麻痺、自律神経失調など）

5 消化器系の問題（胸焼け、胃痛、便秘など）

6 婦人科系の問題（生理不順、生理痛など）

7 泌尿器系の問題（膀胱炎、頻尿、前立腺肥大など）

8 腎臓の問題（腎結石、腎不全など）

9 内分泌系の問題（甲状腺、糖尿病など）

10 皮膚の問題（やけど、皮膚炎、アトピーなど）

11 がん

12 アレルギー

13 慢性的な痛み

14 精神的または心理的な問題（疲労感、ストレス、不眠、うつなど）

15 その他___________________________________________
